# Metagenomic analysis of rhizosphere microflora of oil-contaminated soil planted with barley and alfalfa

**DOI:** 10.1371/journal.pone.0202127

**Published:** 2018-08-09

**Authors:** Vinod Kumar, Sabah AlMomin, Hamed Al-Aqeel, Fadila Al-Salameen, Sindhu Nair, Anisha Shajan

**Affiliations:** Biotechnology Program, Environment and Life Sciences Research Center, Kuwait Institute for Scientific Research, Kuwait City, Kuwait; Oklahoma State University, UNITED STATES

## Abstract

The role of rhizosphere microbial communities in the degradation of hydrocarbons remains poorly understood and is a field of active study. We used high throughput sequencing to explore the rhizosphere microbial diversity in the alfalfa and barley planted oil contaminated soil samples. The analysis of 16s rRNA sequences showed *Proteobacteria* to be the most enriched (45.9%) followed by *Bacteriodetes* (21.4%) and *Actinobacteria* (10.4%) phyla. The results also indicated differences in the microbial diversity among the oil contaminated planted soil samples. The oil contaminated planted soil samples showed a higher richness in the microbial flora when compared to that of untreated samples, as indicated by the Chao1 indices. However, the trend was different for the diversity measure, where oil contaminated barley planted soil samples showed slightly lower diversity indices. While the clustering of soil samples grouped the oil contaminated samples within and across the plant types, the clean sandy soil samples formed a separate group. The oil contaminated rhizosphere soil showed an enrichment of known oil-degrading genera, such as *Alcanivorax* and *Aequorivita*, later being specifically enriched in the contaminated soil samples planted with barley. Overall, we found a few well known oil-degrading bacterial groups to be enriched in the oil contaminated planted soil samples compared to the untreated samples. Further, phyla such as *Thermi* and *Gemmatimonadetes* showed an enrichment in the oil contaminated soil samples, indicating their potential role in hydrocarbon degradation. The findings of the current study will be useful in understanding the rhizosphere microflora responsible for oil degradation and thus can help in designing appropriate phytoremediation strategies for oil contaminated lands.

## Introduction

Microorganisms are probably associated with every living organism on earth. They have been found to play an active role in both animal and plant physiology. Diverse microorganisms live in association with plants, both below and above the ground [1, 2]. They are found within the plants as endophytes and on plant surface as epiphytes. The interaction between the microbial populations can have beneficial and detrimental effects on the plant development and growth [3]. The microbial community residing in the soil has been shown to benefit the plants to a more significant extent. One gram of forest soil contains an estimated 4 X 10^7^ prokaryotic cells [4], which is found to be more than the known catalog of prokaryotes [5]. Soil is one of the major reservoirs of organic carbon, and prokaryotes are an essential part of the soil decomposition system [6]. The biologically active zone of the soil around the plant roots is known as rhizosphere. The rhizosphere harbors soil-borne microbes including bacteria and fungi, which influence the roots through their biological, physical, and chemical interactions. Hence, it is imperative to study the interactions between plants and these soil microorganisms for understanding various plant related processes. However, many groups of microorganisms residing in the soil are not cultivable in the standard laboratory conditions [7]. With the birth of sequencing, and more recently next-generation sequencing (NGS), it has been possible to explore the microbial diversity by using specific genomic regions, such as 16s-rRNA gene. With NGS, it is possible to assess the microbial population from an environmental sample without the need of isolation and culture. This approach termed as metagenomics is a rapidly growing field of microbiology. Metagenomics approach has been used to study the microbial diversity in various contexts in both plants and animals.

The NGS technology has been extensively used to study the human gut microbiome, revealing the interplay between the microorganisms and host metabolism [8, 9]. The approach has also been employed to explore the microbial diversity in plants. In a recent study [10], Mendes et al. used shotgun metagenomics approach to explore the microbial communities in the bulk soil and the rhizosphere of soybean plants, showing an apparent selection at both taxonomic and functional levels. In another study [11], the metagenomic analysis revealed changes in the relative abundance of the bacterial groups, which include strains that promote plant growth and phytic acid utilization, and some genes associated with phytic acid utilization, such as alkaline phosphatase and citrate synthase. Bai et al., [12] assessed the microbial community composition and function in a constructed wetland receiving surface water. The results indicated that the diversity of the rhizosphere soil was found to be significantly higher than that of the wetland influent water, likely due to the availability of diverse habitats and nutrients provided by the wetland plants. The study shows that the use of metagenomic approach can also provide new insights for the study of wetland ecosystems. Metagenomic analysis by Chen et al., [13] showed the importance of microbial population in the phytoremediation of cadmium-contaminated soils. The role of the microbial community has also been established in the degradation of hydrocarbons. A recent study [14] showed the influence of polycyclic aromatic hydrocarbons (PAHs) concentration on the microbial communities in the rice rhizosphere. The microbial population was also affected depending on the distance from the root surface during PAHs degradation. The study also showed that the relative abundance of genes involved in defense mechanisms, replication, and recombination was significantly higher in samples with high PAHs degrading potentials. A study by Koshlaf et al., [15] showed a shift in bacterial communities when pea straw was added to the diesel-contaminated soil. The metagenomic analysis indicated that the original soil contained hydrocarbon degraders (e.g., *Pseudoxanthomonas spp*.), however, treatment with the biostimulant (pea straw) made them active, and accelerated the process of degradation.

Exploring the bacterial communities in aged oil contaminated soil can provide insights into the microbial species involved in bioremediation of oil. Further, different plant species may support different bacterial communities in their rhizosphere as a result of different root exudates. In the current study, we have used NGS technology to explore the microbial communities in different plant species grown in aged oil contaminated soil.

## Materials and methods

### Experimental setup

Seeds of alfalfa cv Regen SY was procured from USDA-WRPIS, Agriculture Research Station, USA. Barley seeds were procured from the local market in Kuwait. The seeds were washed and seeded on moistened potting soil mix and were allowed to germinate and grow for ten days. The seedlings were then transferred to half gallon pots containing aged oil contaminated soil from a site which was contaminated with crude oil during the 90s gulf war. Physical properties of the oil contaminated soil used in the experiments were as follows, pH 7.95±0.01; electrical conductivity 852±17 (μS); salinity 545±11 (ppm); soluble SO_4_ (mg/kg) 4425±218; TPHs 1.33±0.06 (%). Clean sandy-soil from the nearest location of the oil contaminated site was used as a control. Two seedlings were transferred to each pot, and five pots for each treatment were maintained. All the pots were watered daily and fertilized at the end of every two weeks (N:P:K 20:20:20 with trace metals). The plants were maintained in a plant growth chamber set at 25°C and 14:10 light regime. The seedlings were allowed to grow for two months. At the end of two months duration, the plants were removed from the soil and gently shaken to remove excess soil. The rhizospheric soil was collected from the root zone, within five mm from the root surface of the plants from three pots for each treatment using brush and tweezers. Non-rhizospheric control bulk soil samples were also collected for comparative analysis using a cork borer. The soil samples were used for the study of microbial community structure and diversity using 16S rRNA metagenome analysis. DNA was extracted from 0.5 to 2.0 g of the soil using the PowerMax Soil DNA Isolation kit (MoBio, Carlsbad, CA, USA) following manufacturer’s instructions.

### Sequencing of 16s rRNA gene

The 16S rRNA gene variable region V4 PCR primers 515/806 with barcode on the forward primer were used in a 30 cycle PCR (5 cycles used on PCR products) using the HotStarTaq Plus Master Mix Kit (Qiagen, USA) under the following conditions: 94°C for 3 minutes, followed by 28 cycles of 94°C for 30 seconds, 53°C for 40 seconds and 72°C for 1 minute, after which a final elongation step at 72°C for 5 minutes was performed. After amplification, the PCR products were checked in 2% agarose gel to determine the success of amplification and the relative intensity of bands. Multiple samples were pooled together in equal proportions based on their molecular weight and DNA concentrations. Pooled samples were purified using calibrated Ampure XP beads. Then the pooled and purified PCR product was used to prepare DNA library by following Illumina TruSeq DNA library preparation protocol. Sequencing was performed on a MiSeq sequencing platform following the manufacturer’s guidelines. Each sample was sequenced as a paired-end set of reads with a read length of 300 bp. The sequencing was performed at Beijing Genomics Institute (BGI), Hong Kong. The raw data is deposited in NCBI database (SRA: SRP127607; BioProject: PRJNA427666).

### Analysis of high throughput sequencing data

The raw sequence data obtained as paired-end fastq files were checked for quality before and after trimming, using FastQC v0.10.1 tool (https://www.bioinformatics.babraham.ac.uk/projects/fastqc/). The raw data was trimmed for barcode and primer sequences, any ambiguous bases, and homopolymers >6 bases. Reads shorter than 150 bp and with an average Phred quality score of <20 were removed. All the trimming and filtering steps were performed using Quantitative Insights Into Microbial Ecology (QIIME) version 1.9.0 [16].

The trimmed sequences were checked for chimeric sequences by using both *de-novo* and reference based methods. The RDP Gold database v9 reference was used for the reference based detection of chimeric sequences using USEARCH program [17]. The detected chimeric sequences were removed and only the non-chimeric sequences were considered for further analysis. The chimera filtered sequences were used for the identification of operational taxonomic unit (OTU) clusters with a minimum similarity of 97% using UCLUST [17], and the clusters were used for generating representative sequences. The representative sequences were further filtered to remove any singletons using QIIME. The filtered representative sequences were aligned using PyNAST [18], a method for performing pair-wise alignment. The alignment file was further filtered for positions with gaps, and outliers (sequences dissimilar to the alignment consensus). The filtered representative sequences were mapped against the greengenes database [19] with a similarity of 80% using Ribosomal Database Project (RDP) classifier [20]. All the data analysis steps were performed using the tools implemented within QIIME [16].

Filtered alignments were considered for various statistical analysis and phylogenetic tree construction. Alpha diversity representing the diversity and richness of each sample was calculated by rarefying a small percentage of randomly picked sequences, and considering 10 iterations each time. The rarefaction analysis was performed by considering the sampling depth of 55,000 sequences per sample. The Shannon index indicating the diversity and Chao1 index indicating the richness of microbial population were calculated using the taxonomic classifications and phylogenetic tree. Beta diversity indicating the diversity across samples was calculated using the weighted and unweighted UniFrac [21] distance matrix. Principal Coordinate Analysis (PCoA) was performed using the UniFrac results. All the statistical analyses were performed using QIIME tool [16]. Significant differential abundance of taxonomic assignments across samples was identified using t-test. Phyla and genera having a p-value of < 0.05 were considered as significantly enriched.

Unweighted Pair Group Method with Arithmetic mean (UPGMA) clustering was performed using the biom table in QIIME. Heatmap was generated using METAGENassist [22], with genus level counts across samples. Reads that were unassigned or unmapped, and the genus with over 50% zeros (across samples) were excluded while generating the heatmap.

## Results

### Quality analysis, raw data trimming, and chimera detection

The sequencing of 16s r-RNA gene from 15 samples resulted in 1,628,728 reads. The paired-end raw data obtained in fastq files was subjected to stringent trimming and filtering. Around 91% of the data was retained after various quality filtering steps, **Table 1**. The counts of filtered reads per sample after the quality filtering are shown in **Table 2**. Approximately 3% of the raw reads were removed because of the mismatches in primer sequence. A total of 66,414 chimeric sequences corresponding to 4.08% of the raw reads were detected and excluded from further analysis, **Table 3**. Reference based chimera analysis detected more number of chimeric sequences than the *de-novo* method.

**Table 1:** Summary of read count after various filtering steps.

Filtering criteria	Number of reads	Percentage of reads
Length outside bounds of 150 and 1000	290	0.02
Ambiguous bases exceeds limit of 0	0	0.00
Missing quality score	0	0.00
Mean quality score below minimum of 20	0	0.00
Max homopolymer run exceeds limit of 6	21,363	1.31
Mismatches in primer exceeds limit of 0	56,136	3.45
Chimeric sequences	66,414	4.08
**Total removed**	**144,203**	**8.85**
**Total retained**	**1,484,525**	**91.15**

**Table 2:** Summary of raw and quality filtered reads per sample.

Sample ID	No. of raw reads	No. of reads after filtering	Percentage retained	Percentage removed
CS_R1	108,687	102,478	94.29	5.71
CS_R2	78,990	75,446	95.51	4.49
CS_R3	77,801	72,750	93.51	6.49
BC_R1	104,458	100,389	96.10	3.90
BC_R2	85,042	81,632	95.99	4.01
BC_R3	94,548	91,020	96.27	3.73
BO_R1	107,481	102,599	95.46	4.54
BO_R2	119,154	114,554	96.14	3.86
BO_R3	119,167	113,237	95.02	4.98
AC_R1	121,854	117,154	96.14	3.86
AC_R2	99,215	94,663	95.41	4.59
AC_R3	183,420	176,056	95.99	4.01
AO_R1	102,121	97,991	95.96	4.04
AO_R2	109,799	105,793	96.35	3.65
AO_R3	116,991	112,508	96.17	3.83
**Total**	**1,628,728**	**1,558,270**	**95.67**	**4.33**

CS: Clean sandy soil; BC: Control oil contaminated bulk soil without barley plants; BO: Barley planted oil contaminated rhizosphere soil; AC: Control oil contaminated bulk soil without alfalfa plants; AO: Alfalfa planted oil contaminated rhizosphere soil.

**Table 3:** Summary of chimera detection and filtering.

	No. of reads
Reference non chimeras	1,411,677
Reference chimeras	146,593
*De-novo* chimeras	110,608
*De-novo* non chimeras	1,447,662
Total chimeras	66,414
Total reads after filtering chimeras	1,491,856
**Percentage retained (of filtered reads)**	**95.74**

### OTU generation, filtering and alignment

The filtered reads were grouped into OTU clusters. A total of 154,254 OTUs were identified across all the samples, of which 29,894 were retained after filtering the singletons. Singleton OTUs are the clusters containing only one read sequence. The filtered OTUs represented 1,368,497 sequences from all samples, corresponding to an average number of 91,233 sequences per sample, **Table 4**. A single representative sequence was obtained from each OTU cluster and subjected to alignment. All the representative sequences except 69 produced alignment hits. After filtering alignments with gaps, and removing outliers, 29,630 aligned sequences were obtained.

**Table 4:** Number of reads per sample after filtering singleton OTUs.

Sample ID		No. of reads assigned to OTUs after filtering singletons
CS_R1		85,062
CS_R2		61,993
CS_R3		59,345
BC_R1		88,024
BC_R2		72,434
BC_R3		79,711
BO_R1		91,975
BO_R2		102,965
BO_R3		100,921
AC_R1		103,214
AC_R2		84,141
AC_R3		156,630
AO_R1		88,212
AO_R2		93,209
AO_R3		100,661
**Total sequences/reads**		**1,368,497**

CS: Clean sandy soil; BC: Control oil contaminated bulk soil without barley plants; BO: Barley planted oil contaminated rhizosphere soil; AC: Control oil contaminated bulk soil without alfalfa plants; AO: Alfalfa planted oil contaminated rhizosphere soil.

### Taxonomic classification

**Fig 1:**
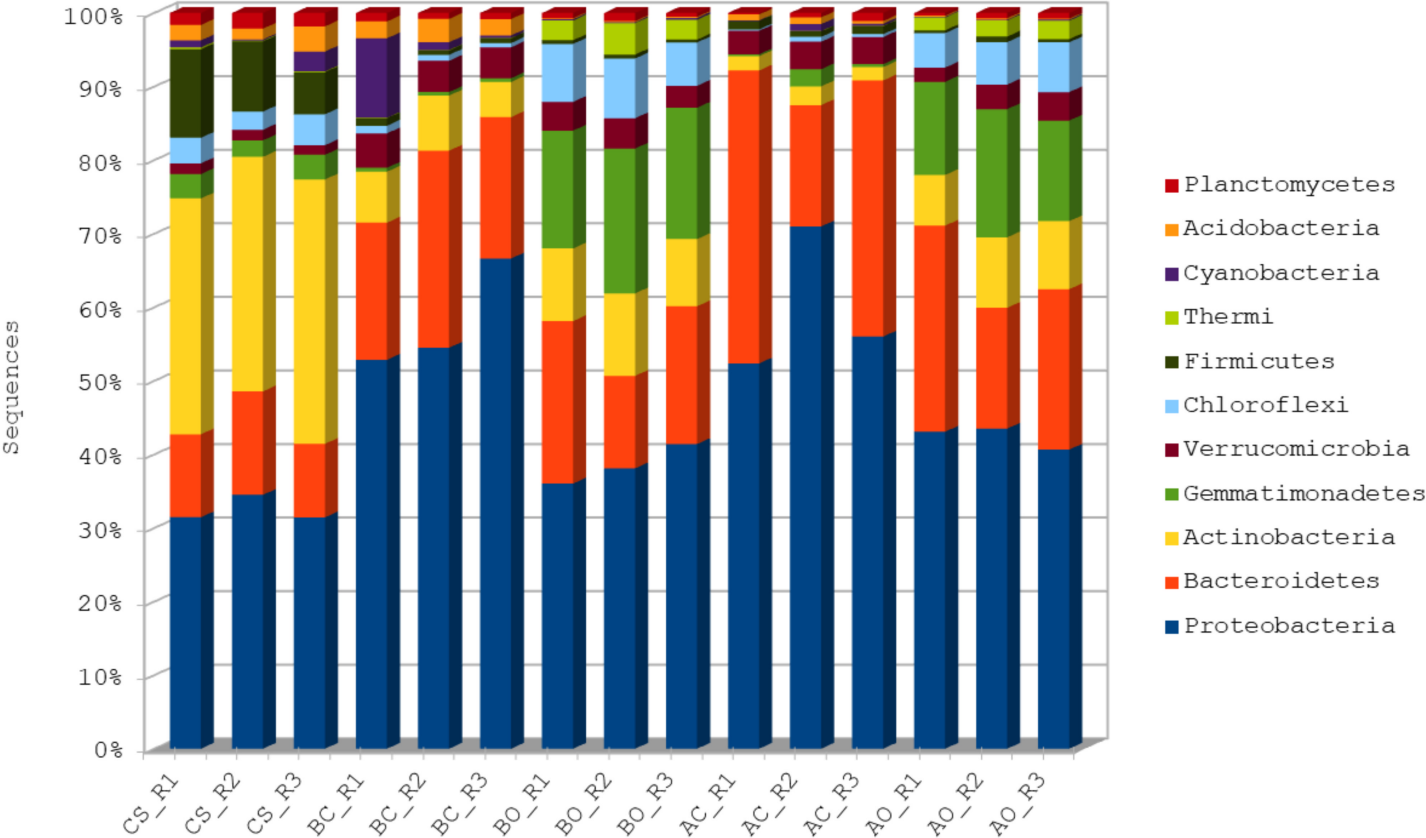
Phylum level distribution of microbial population across samples. Phyla represented by at least 0.5% of the total assigned sequences (11 of total 36 phyla) are shown here. CS: Clean sandy soil; BC: Control oil contaminated bulk soil without barley plants; BO: Barley planted oil contaminated rhizosphere soil; AC: Control oil contaminated bulk soil without alfalfa plants; AO: Alfalfa planted oil contaminated rhizosphere soil.

Around 1.3 million sequences were assigned to various taxa with at least 80% similarity. A total of 36 taxa at phylum level were identified across samples, of which 11 are represented by at least 0.5% of total reads. Phylum level distribution across samples for these 11 taxa is presented in **Fig 1.** The distribution of all the phyla can be found in **S1 File**. Major percentage of the sequences were assigned to *Proteobacteria* (45.9%), followed by *Bacteriodetes* (21.4%) and *Actinobacteria* (10.4%). However, we found bacterial population from the phylum *Proteobacteria* to be comparatively low in the clean sandy soil (common control) group. Ninety seven percent of the total sequences were assigned to the top 10 phyla, **S1 File**. The oil contaminated soil showed a decreased population of *Proteobacteria* compared to the untreated soil, in both the plants groups. The clean sandy soil was found to be enriched with the bacterial population from the phylum *Actinobacteria*. Further, the oil contaminated soil sample showed a slight increase in the microbial population from the phylum *Actinobacteria* compared to the clean soil samples, **Fig 1**. The microbial population from the phylum *Gemmatimonadetes* showed enrichment specifically in the oil contaminated planted soil samples. However, *Firmicutes* were shown to be specifically enriched in the clean sandy soil samples.

**Fig 2:**
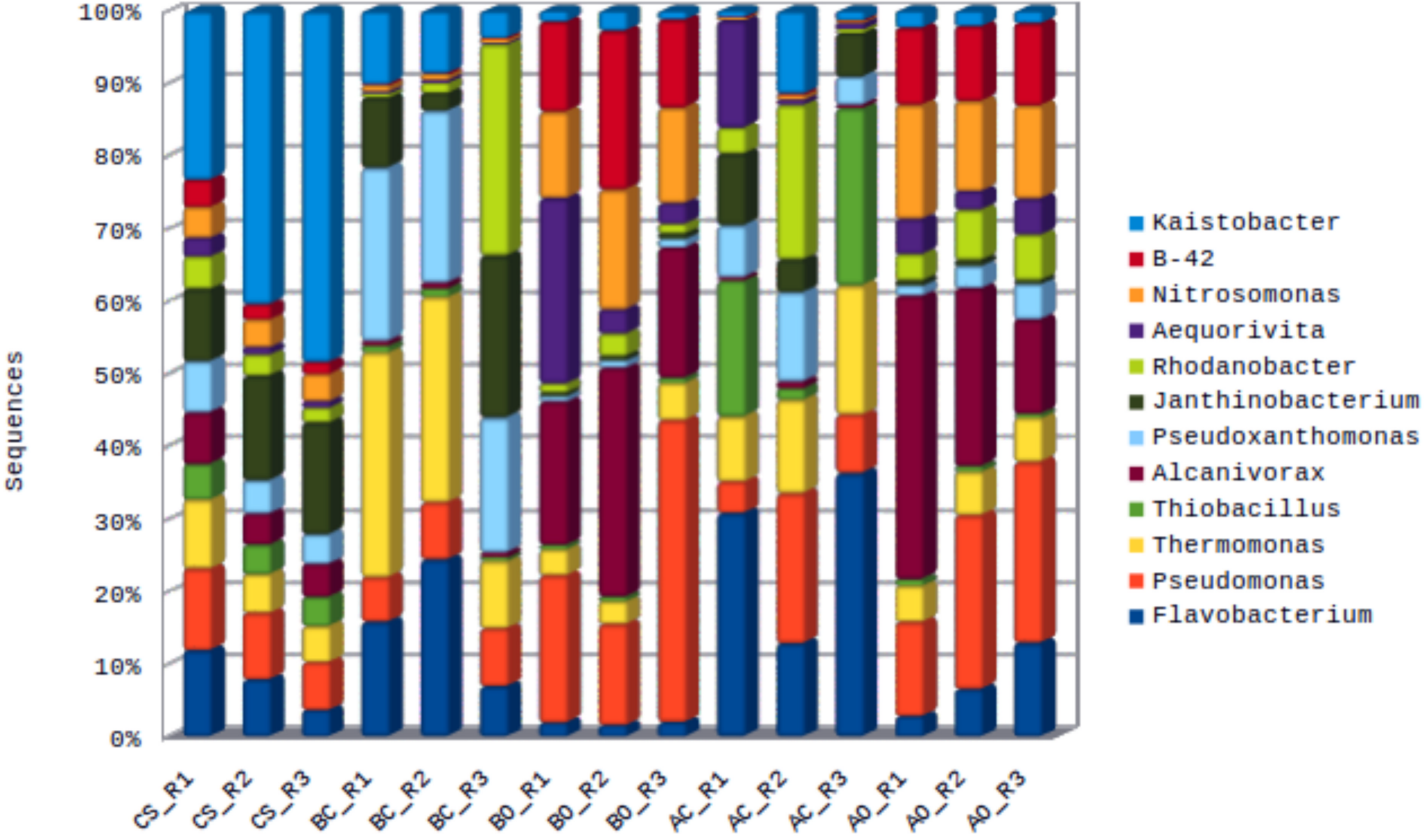
Genus level distribution of microbial population across samples. Genera with at least 2% of the total assigned sequences (12 of total 372 genera) are represented here. CS: Clean sandy soil; BC: Control oil contaminated bulk soil without barley plants; BO: Barley planted oil contaminated rhizosphere soil; AC: Control oil contaminated bulk soil without alfalfa plants; AO: Alfalfa planted oil contaminated rhizosphere soil.

A total of 657,190 sequences were assigned to 372 genera. There were 26 genera covering 72% of the total assigned sequences and represented by at least 1% of the total assigned sequences. *Flavobacterium* was the most enriched genus among the classified genera, representing 9.6% of the total assigned sequences, followed by *Pseudomonas* (7.0%) and *Thermomonas* (6.3%). Genus level distribution for the taxa with at least 2% abundance is shown in **Fig 2**. The distribution of all the genera can be found in **S1 File**. The distribution of *Pseudomonas* across the oil contaminated soil samples planted with barley or alfafa was shown to be comparatively more than in the untreated or clean sandy soil samples. In contrast, the distribution of *Flavobacterium* was found to be decreased comparatively in the oil contaminated soil samples than in the untreated groups. We found similar correlation with the microbial population from the genus *Thermomonas*. The oil contaminated soil samples planted with barley and alfalfa were found to be specifically enriched in the genera *Alcanivorax*, *Nitrosomonas* and *B-42* (**Fig 2**).

Significance enrichment analysis also indicated a difference in the distribution of microbial population across the treated and untreated groups, with a p-value <0.05, **Table 5** and **S2 File**. The phyla *Gemmatimonadetes* (p-value 5.6E-05), *BRC1* (p value 6.99E-05) and *Chloroflexi* (p-value 1.72E-04) were the top 3 that showed significant differences between the oil contaminated soil planted with alfalfa or barley and the untreated soil samples. Bacteria from the phyla *Armatimonadetes*, *TM6*, and *TM7* were found to be significantly enriched only in barley planted oil contaminated soil samples compared to the untreated. *SBR1093* and *Armatimonadetes* were found to be the most significant phyla in oil contaminated soil samples planted with alfalfa and barley respectively compared to their untreated counterparts. Interestingly, microbial population from a few phyla were also found to be enriched in all the planted soil samples compared to the clean sandy soil samples, with *Verrucomicrobia* being the most significant.

**Table 5:** Significantly enriched phyla between planted and untreated oil contaminated soil samples.

Enriched phylum	Enrichment P-value
Gemmatimonadetes	5.6E-05
BRC1	7.0E-05
Chloroflexi	1.7E-04
Actinobacteria	2.9E-04
SBR1093	3.2E-04
Thermi	1.2E-03
Acidobacteria	9.0E-03
Nitrospirae	2.9E-02
Armatimonadetes	3.2E-02
TM6	3.2E-02
WS6	4.2E-02

The enrichment analysis was performed by combining both oil contaminated soil samples planted with alfalfa and barley versus all the untreated samples.

Among the 372 genera compared, *Halorhodospira* was found to be the most significantly (p-value 2.30E-06) enriched in planted oil contaminated soil group compared to the untreated soil sample group. When the planted oil contaminated and untreated soil sample groups were compared with the clean sandy soil sample group, *Rubellimicrobium* was found to be the most significantly enriched genus.

### Analysis of sample diversity and clustering

**Fig 3:**
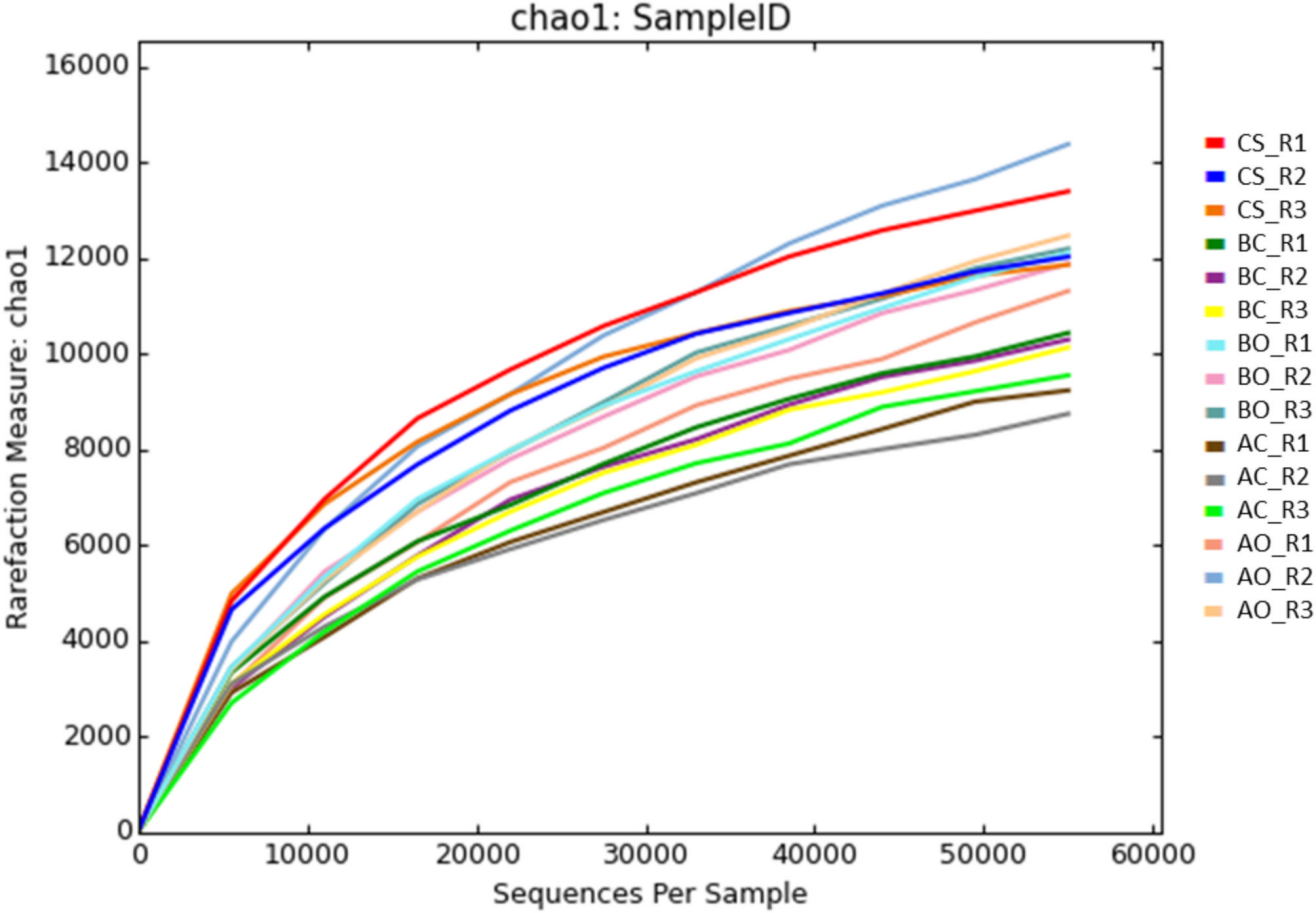
Species richness indicated by Chao1 rarefaction measure. CS: Clean sandy soil; BC: Control oil contaminated bulk soil without barley plants; BO: Barley planted oil contaminated rhizosphere soil; AC: Control oil contaminated bulk soil without alfalfa plants; AO: Alfalfa planted oil contaminated rhizosphere soil.

The microbial diversity of different soil samples was studied using Alpha and Beta diversity indices. Alpha diversity indices are used to estimate the diversity within a sample, whereas, Beta diversity is used to estimate the microbial diversity across the communities or samples. Chao1 and Shannon indices were calculated using the rarefaction sampling to estimate the Alpha diversity of different soil samples. The Chao1 index is commonly used to estimate the species richness of a sample, and is based upon the number of rare classes (i.e. OTUs) found in a sample, **Fig 3** and **Table 6**. Overall, clean sandy soil samples showed the highest richness. The species richness was also shown to be increased with increasing number of sequences for all the soil samples. The Chao1 index overall indicated a slight increase in the richness of species in oil contaminated samples planted with alfalfa or barley compared to the untreated sample groups.

**Table 6:** Shannon and Chao1 indexes for each sample.

Sample ID		Shannon index	Chao1 index
CS_R1		9.0	9369.5
CS_R2		9.0	8507.8
CS_R3		9.2	8656.2
BC_R1		7.7	6951.6
BC_R2		7.6	6800.7
BC_R3		7.5	6692.2
BO_R1		7.0	7940.1
BO_R2		7.0	7798.1
BO_R3		6.9	8027.6
AC_R1		6.9	6090.8
AC_R2		7.6	5915.5
AC_R3		6.7	6302.2
AO_R1		6.8	7248.3
AO_R2		7.5	9337.5
AO_R3		7.3	8037.7

CS: Clean sandy soil; BC: Control oil contaminated bulk soil without barley plants; BO: Barley planted oil contaminated rhizosphere soil; AC: Control oil contaminated bulk soil without alfalfa plants; AO: Alfalfa planted oil contaminated rhizosphere soil.

**Fig 4:**
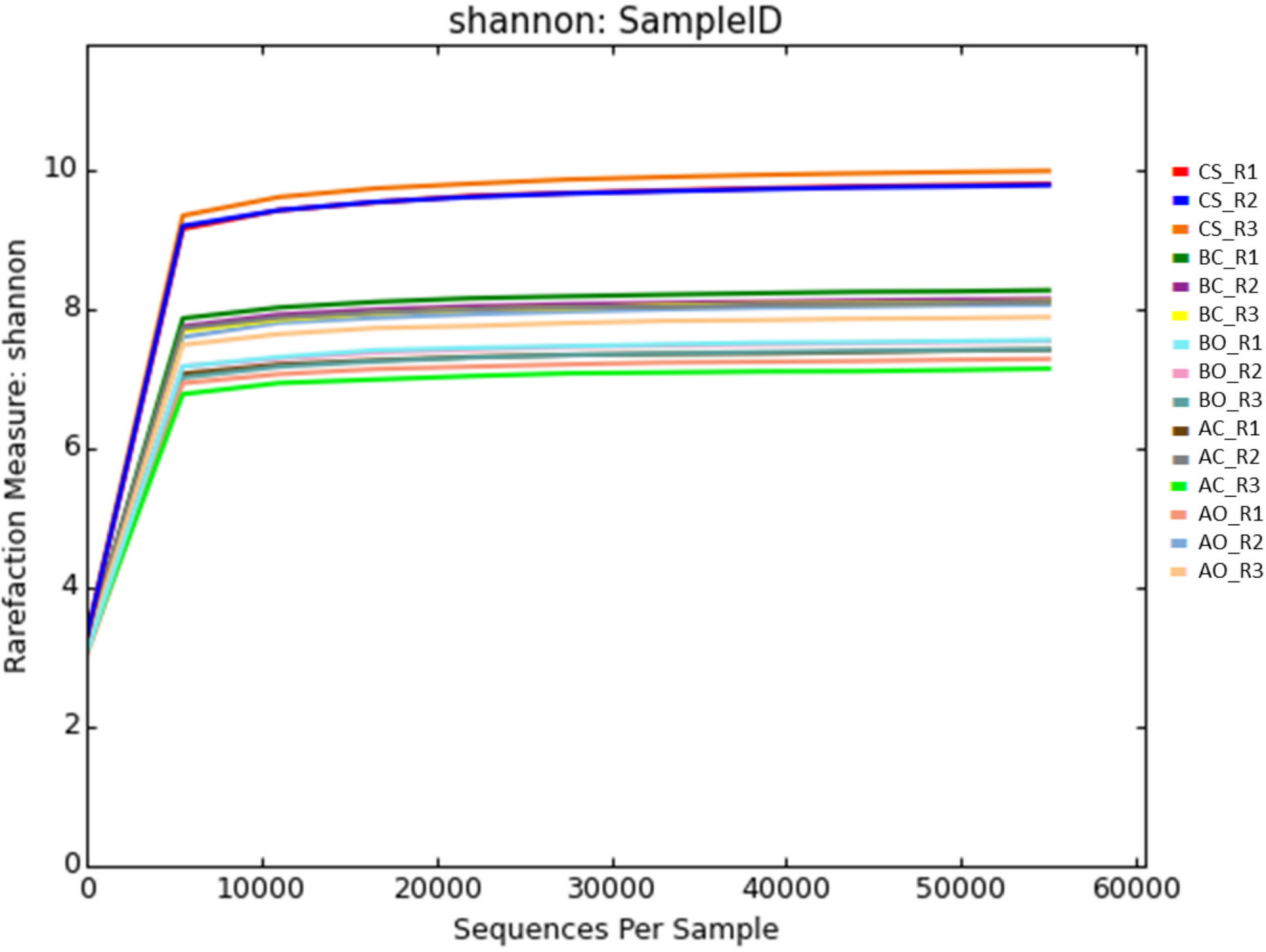
Species diversity estimated by Shannon index. CS: Clean sandy soil; BC: Control oil contaminated bulk soil without barley plants; BO: Barley planted oil contaminated rhizosphere soil; AC: Control oil contaminated bulk soil without alfalfa plants; AO: Alfalfa planted oil contaminated rhizosphere soil.

The Shannon index used to estimate the sample diversity indicated enough sampling depth at 12,000 sequences, **Fig 4**. The microbial diversity was found to be highest for the clean sandy soil samples, similar to sample richness index, **Table 6**. The diversity of the oil contaminated samples planted with barley was lower compared to the untreated soil samples, which was found to be opposite to the Chao1 richness index. For alfalfa planted soil samples the difference between the planted and untreated control was shown to be negligible.

**Fig 5:**
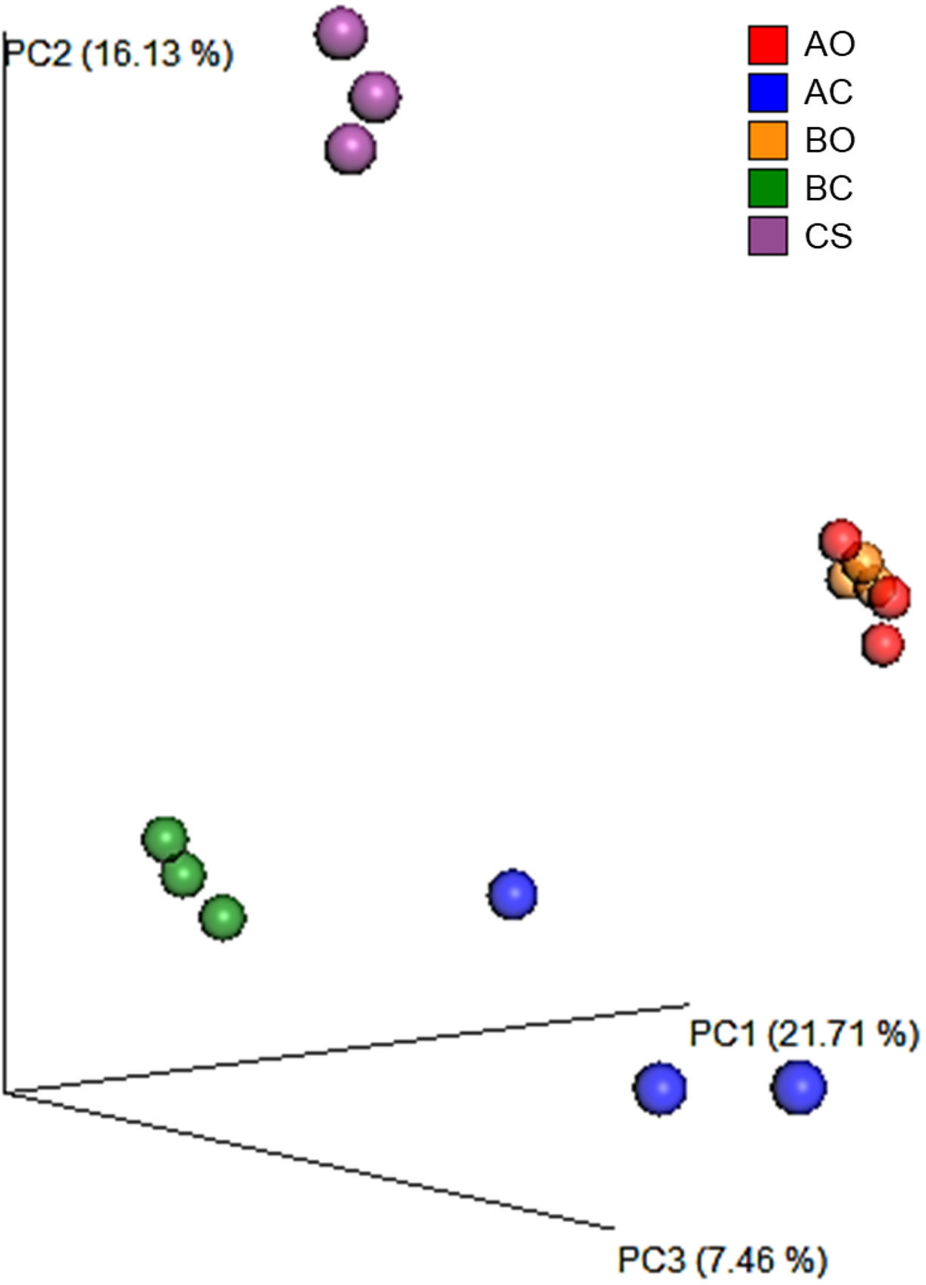
PCoA plot of samples using unweighted UniFrac analysis. CS: Clean sandy soil; BC: Control oil contaminated bulk soil without barley plants; BO: Barley planted oil contaminated rhizosphere soil; AC: Control oil contaminated bulk soil without alfalfa plants; AO: Alfalfa planted oil contaminated rhizosphere soil.

**Fig 6:**
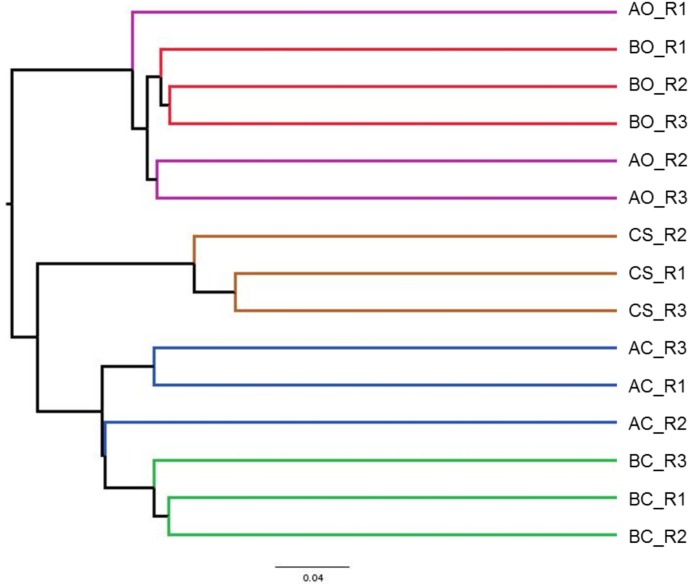
UPGMA clustering of samples based on phylogenetic distribution. CS: Clean sandy soil; BC: Control oil contaminated bulk soil without barley plants; BO: Barley planted oil contaminated rhizosphere soil; AC: Control oil contaminated bulk soil without alfalfa plants; AO: Alfalfa planted oil contaminated rhizosphere soil.

**Fig 7:**
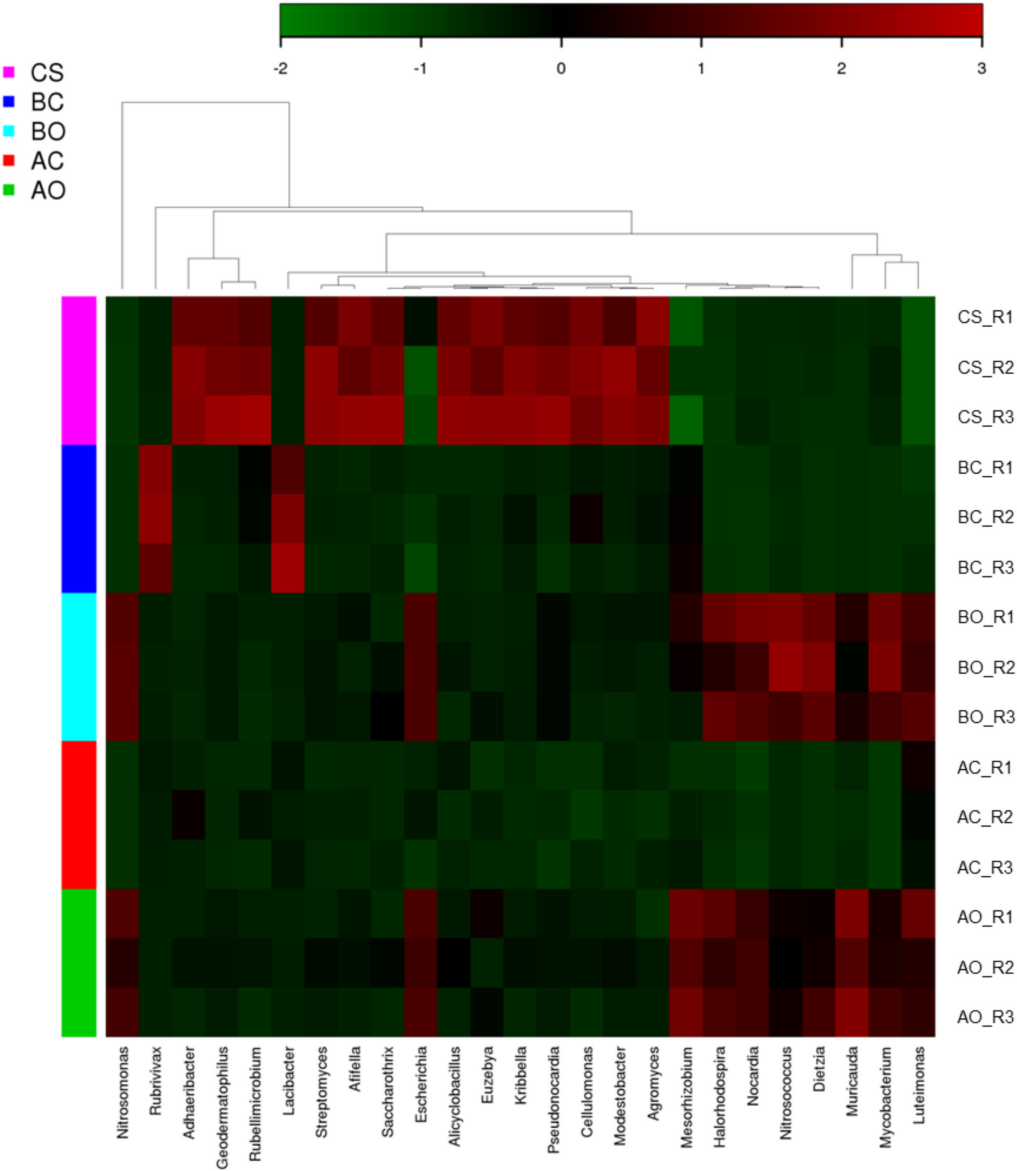
Heatmap representing the genus-level distribution of bacterial population across samples. CS: Clean sandy soil; BC: Control oil contaminated bulk soil without barley plants; BO: Barley planted oil contaminated rhizosphere soil; AC: Control oil contaminated bulk soil without alfalfa plants; AO: Alfalfa planted oil contaminated rhizosphere soil.

Beta diversity was used to estimate the sample diversity across the soil samples. To estimate the beta diversity, weighted and unweighted UniFrac distance matrix was used. PCoA plots using the unweighted UniFrac results indicated that the samples from same group clustered together, **Fig 5**. The oil contaminated soil samples planted with barley and alfalfa plants clustered together indicating the existence of common microorganisms. Clean sandy soil samples as expected formed a separate cluster indicating a different microbial diversity compared to other soil samples. These results were further corroborated by the UPGMA clustering, **Fig 6**. Heatmap using the top 25 enriched genera showed a similar pattern in oil contaminated soil samples planted with barley and alfalfa plants, **Fig 7**. Genera such as, *Mycobacterium*, *Nocardia*, and *Halorhodospira* were shown to be specifically enriched in these sample groups. Clean sandy soil clearly showed a distinct enrichment of genera such as, *Afifella*, *Euzebya*, *Geodermatophilus*, and *Modestobacter*. We found enrichment of genera *Lacibacter* in rhizosphere soil samples planted with barley plants. Genus *Mesorhizobium* was found to be specifically enriched in the oil contaminated soil samples planted with alfalfa.

## Discussion

The plant rhizosphere actively secretes a number of compounds that are used by the microbial communities present in the soil around the plant roots. These microorganisms help in the growth and development of the plants. Contamination of the soil with different contaminants, such as petroleum hydrocarbons may affect the microbial composition of the rhizosphere, which in turn may have adverse effects on the plant development. The use of plants in the removal of contaminants, also called as phytoremediation, has been a subject of interest among many microbiologists. Microorganisms involved in hydrocarbon degradation are well-studied microbial groups. Many bacterial, algal, and fungal genera indeed have been recognized as capable hydrocarbon degraders [23, 24]. In the current study, we used high throughput sequencing technology to explore the microbial diversity of oil contaminated soil planted with barley and alfalfa plants. We compared the microbial composition of the oil contaminated soil planted with barley and alfalfa with their untreated counterparts and clean sandy soil to understand the effect of aged crude oil on the microbial diversity of rhizosphere. The analysis clearly differentiates the microbial composition and abundance between both the soil sample groups. Study by Lupatini et. al., [25], showed that the soil samples of different farming systems are dominated by phyla such as, *Proteobacteria*, *Bacteroidetes* and *Actinobacteria*. The soil samples in the current study, irrespective of the treatments showed a similar microbial dominance of these phyla. However, the abundance of *Proteobacteria* and *Bacteroidetes* was comparatively more in the untreated oil contaminated soil samples in case of both the plant types.

Alfalfa plant has been used to remove contaminants in many studies [26, 27]. Kim et. al., [26] explored the rhizosphere of diesel-contaminated soils planted with alfalfa, and showed that the total microbial activity was highest in diesel-contaminated rhizosphere soils. Further, significantly more hydrocarbon-degraders were found in diesel-contaminated rhizosphere soil compared to unplanted and uncontaminated soil. Our study showed similar results for both barley and alfalfa planted rhizosphere soil samples. Oil contaminated barley planted soil showed an average increase of 45 fold, and alfalfa planted soil showed an average increase of 40 fold for the bacterial species from genus *Alcanivorax*, a known oil degrader. A slight increase in microbial strains of *Pseudomonas* was also seen in the barley planted oil contaminated soil samples. Muratova et. al., [27] showed that the changes in the microbial community under bitumen contamination does not depend only on the presence of the plant, but also on the type of plant. They also showed that the rhizosphere microflora of alfalfa had a higher degradative potential. However, our study showed a higher increase in the number of hydrocarbon degraders in the barley planted soil. The genus *Aequorivita* has also been proposed to have hydrocarbon degradation potential in culture media [28]. Our study showed around 52 fold enrichment of bacterial species from *Aequorivita* genus in the barley planted oil contaminated soil compared to the untreated samples, indicating the degradative potential of the genus in barley rhizosphere. However, the same genus was shown to be decreased by around 2 fold in the alfalfa planted oil contaminated soil. *Nitrosomonas*, a nitrifying bacterial genus was found to be enriched by more than 25 fold in both barley and alfalfa planted oil contaminated soil samples indicating its possible role in oil degradation. A low abundant phylum *Thermi* (~1.2%) showed higher fold enrichment in the oil contaminated soil samples compared to the control clean soil. In contaminated soil planted with alfalfa and barley, the enrichment was shown to be 59 and 67 fold respectively compared to their untreated counterparts. Genus *B-42* from the same phylum showed a high fold enrichment of around 150 in the oil contaminated planted soil samples compared to the untreated soil samples. A few studies [29, 30] have also shown the presence of *Deinococcus*, a class of *Thermi* group, in the oil contaminated samples, however their role in oil degradation has not been established. A study by An et. al., [31] showed the role of organisms from phylum *Chloroflexi* in anaerobic hydrocarbon degradation. Our study indicated around 11 fold increase in the population of this phylum in the oil contaminated planted soil samples compared to the untreated samples.

Our study explored the microbial diversity of clean desert soil and untreated oil contaminated soil with oil contaminated rhizosphere soil planted with barley and alfalfa plants. We observed differences in the diversity and enrichment of microflora in the planted soil samples compared to the untreated samples. Further, the results showed variation based on the type of plant used. We identified a few known oil degrading bacterial genera, such as *Alcanivorax* and *Aequorivita* to be enriched in the oil contaminated planted soil samples. *Gemmatimonadetes*, a well abundant phyla across the samples, was found to be significantly enriched (p value 5.6E-05), with a high fold increase (average fold of ~25) in the oil contaminated planted soil samples compared to the untreated counterparts. A similar trend was observed with a low abundant phylum *Thermi*, which showed significant enrichment (p value 0.001239) with an increase of 63 fold on an average. Though a casual association of these phyla with hydrocarbon degradation cannot be ruled out, their exact role in the oil remediation needs to be evaluated further. The findings of the current study will be useful in understanding the microbial population responsible for oil degradation, and hence can be helpful in designing appropriate phytoremediation strategies for oil contaminated lands.

## Supporting information

S1 File Distribution of the microbial population at phylum and genus level across all the sequenced samples. Number indicates the read count for a phylum/genus in each sample.
(XLS)

S2 File Statistically significant (student t-test, p <0.05) phylum and genus across the sample groups. (XLS)
